# The Burden of Musculoskeletal Conditions

**DOI:** 10.1371/journal.pone.0090633

**Published:** 2014-03-04

**Authors:** Clémence Palazzo, Jean-François Ravaud, Agathe Papelard, Philippe Ravaud, Serge Poiraudeau

**Affiliations:** 1 U1153, Institut National de la Santé et de la Recherche Médicale, Paris, France; 2 Centre d'Épidémiologie Clinique, Hôpital Hôtel Dieu AP-HP, Paris, France; 3 Université Paris Descartes, PRES Sorbonne Paris Cité, Paris, France; 4 Service de rééducation et réadaptation de l'appareil locomoteur et des pathologies du rachis, Hôpital Cochin AP-HP, Paris, France; 5 Institut fédératif de recherche sur le handicap, Institut National de la Santé et de la Recherche Médicale, Paris, France; 6 U988, Institut National de la Santé et de la Recherche Médicale, Villejuif, France; 7 UMR 8211, Centre national de la recherche scientifique, Villejuif, France; Center for Rheumatic Diseases, India

## Abstract

**Objective:**

Despite the burden of rheumatic and musculoskeletal diseases (RMDs), these conditions probably deserve more attention from public health authorities in several countries including developed ones. We assessed their contribution to disability.

**Methods:**

Data on disabilities associated with RMDs were extracted from the national 2008–2009 Disability-Health Survey of 29,931 subjects representative of the population in France. We used the core set of disability categories for RMDs of the World Health Organization's International Classification of Functioning, Disability and Health for analysis. Diagnosis and disabilities were self-reported. We assessed the risk of disability associated with RMDs using odds ratios (ORs) and the societal impact of RMDs using the average attributable fraction (AAF).

**Results:**

Overall 27.7% (about 17.3 million people) (95% CI 26.9–28.4%) of the population reported having RMDs. The most prevalent RMDs were low back pain (12.5%, 12.1–13.1) and osteoarthritis (12.3%, 11.8–12.7). People reporting osteoarthritis were more disabled in walking (adjusted OR 1.9, 1.7–2.2) than those without. People reporting inflammatory arthritis were more limited in activities of daily living (from 1.4, 1.2–1.8 for walking to 2.1, 1.5–2.9 for moving around). From a societal perspective, osteoarthritis was the main contributor to activity limitations (AAF 22% for walking difficulties). Changing jobs was mainly attributed to neck pain (AAF 13%) and low back pain (11.5%).

**Conclusion:**

RMDs are highly prevalent and significantly affect activity limitations and participation restrictions. More effort is needed to improve care and research in this field.

## Introduction

Rheumatic and musculoskeletal diseases (RMDs) are a major cause of disability [1]. In the 2010 World Health Organization Global Burden of Disease (WHO-GBD) study, low back pain (LBP) was the leading cause of years lived with disability in the world, neck pain the fourth cause, and other musculoskeletal disorders the fifth; osteoarthritis (OA) increased from 15th in 1990 to 11th in 2010 in western Europe [1]. RMDs affect individuals by limiting their activities and restricting their participation [2] and affect societies by work loss, disability pensions, early retirement and the increasing need for social support [3], [4].

Despite the burden of RMDs and the WHO's Joint and Bone decade initiative extension until 2020 [5], these conditions probably deserve more attention from public health authorities in several countries including developed ones. A barrier for prioritization of RMDs by public health policymakers is that they are not considered to be fatal, even though OA has been shown to be associated with increased mortality [6]. Prioritization has an impact on health system performances. For example, the French health system, where research and care for cardiovascular risks have been prioritized, perform significantly better than the mean of 18 other health systems for stroke, ischemic heart disease and diabetes but quite poorly for RMDs such as neck and back pain or osteoarthritis [7]. Production of representative national data on disability with a focus on RMDs may help national policymakers to prioritize public health strategies and convince them of the need to consider focusing on musculoskeletal conditions to improve population health.

Studies describing disabilities in RMDs are common, but they often focus on one condition, such as rheumatoid arthritis or OA [8], [9], and definitions used for disability vary substantially, making comparison between data difficult. The International Classification of Functioning, Disability and Health (WHO-ICF), the WHO's framework for measuring health and disability, provides a worldwide accepted framework, with specific core sets developed and validated for musculoskeletal conditions [10]–[14]. These core sets, which are short lists of WHO-ICF categories relevant to specific diseases, serve as practical tools for clinical practice and allow for standardization of data for health information and research [13], [15].

A population-based, self-reported disability survey, the 2008–2009 Disability-Health (DH) Survey, was conducted in France. We previously extracted data from this dataset to assess the respective contribution of chronic conditions to disability in the non-institutionalized population and showed that RMDs, neurological, cardiovascular, and psychiatric disorders were the main contributors [2]. Here, we aimed to assess the contribution of different RMDs to WHO-ICF disability categories at the individual and societal levels.

## Methods

### Ethics

This study was planned as a research project. It was performed in collaboration with the French National Institute of Statistics. This study was declared of public interest by the CNIS (Conseil National d'Information Statistique) and was approved by the CNIL (Commission Nationale de l'Informatique et des Libertés, French law no. 78-17). According to the French law, written informed consent was not required for this type of study.

### Disability-health (DH) survey

**Figure 1:**
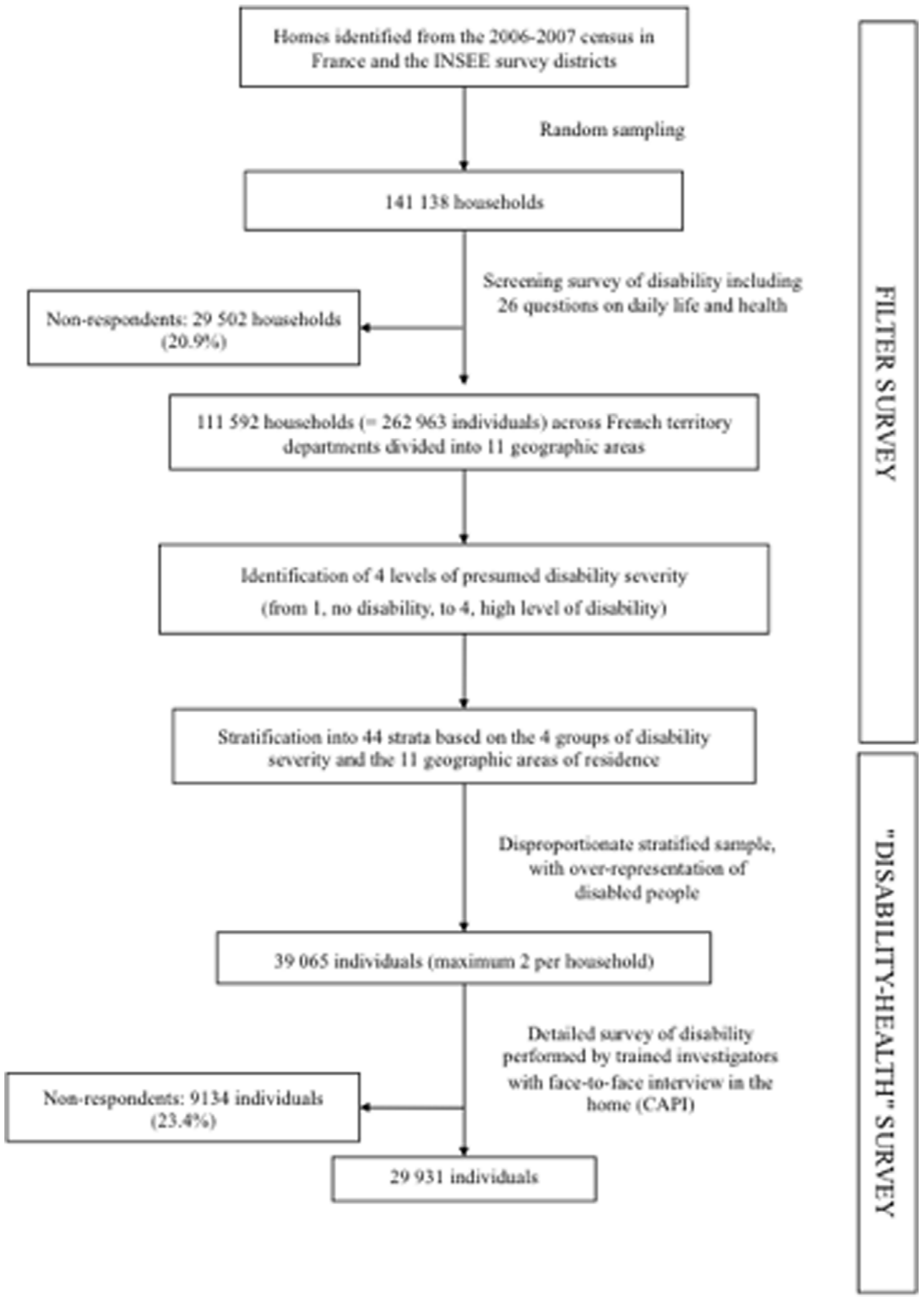
Design of the representative national “Disability-Health” survey. INSEE  =  French National Institute of Statistics and Economic Studies.

The data for this study were from the 2008–2009 DH survey (available at http://www.cmh.ens.fr/greco/enquetes/XML/lil-0459.xml), a cross-sectional population-based survey undertaken by the French National Institute of Statistics and Economic Studies and the French Head Office of Research, Studies, Evaluation and Statistics of the Social Affairs Ministry to describe disabilities. The present work focused on the part of the survey involving the population living in a household (**Figure 1**).

The DH survey methodology has been described in detail elsewhere [2]. Briefly, a two-stage method was used. First, a preliminary filter-survey was conducted to identify people who were disabled. According to their answers to the filter survey, people were classified into four levels of presumed disability severity, from 1 (no disability) to 4 (high level of disability). The survey also involved intensive sampling in several geographic areas to obtain representative data in these areas. Stratification into 44 strata was based on the four presumed disability levels and the 11 geographic areas of residence.

Second, for DH sample selection, randomisation involved a high sampling rate for the most severely disabled group (in order to include people who are disabled, and so to have enough data on disability) and a low sampling rate for people without daily living restrictions (the largest group). Consequently, the survey respondents were not representative of the French population, but a weighting method, described in the statistic analysis section, allows for estimating representative results at a national level: each of the resulting groups was allocated a specific sampling coefficient that increased with the probability or severity of the presumed handicap. The sampling rate was higher for people living in the geographic areas that were more intensively sampled. From March to July 2008, data were collected for 39 065 subjects across the administrative departments in France. Trained investigators used the computer-assisted interview (CAPI) format to collect data from people in their homes. The questionnaire was 160 pages long and each interview lasted between 2 and 3 hours. A household member or a proxy could answer for identified survey respondents not able to answer alone. The response rate was 76.6%, corresponding to 29 931 subjects with complete data (age range 0–106 years old). Each respondent was assigned a weight reflecting the probability of being investigated and answering the questionnaire, which allowed for estimating representative results at a national level.

### Definition of rheumatic and musculoskeletal disorders (RMDs)

RMDs were self-reported. Participants were asked to identify their diseases from a list of 52 disorders, which is known to improve the accuracy of self-reporting [16]. Among the disorders, six musculoskeletal diseases were identified: OA (knee, hip and other OA), low back pain, neck pain, inflammatory arthritis (including rheumatoid arthritis), spine deformity, and osteoporosis.

The survey also collected data on co-morbidities with the same list, which included cardiovascular, neurological, psychiatric, respiratory, sensorial, endocrine, digestive, urologic, dermatologic disorders, and sequelae of injury (**Table S1**), as a numerical variable (number of co-morbidities) or dichotomous variable (co-morbidity represented at least one of these conditions). Data on sex, age, and educational attainment (no diploma represented less than primary school education) were also collected.

### Definition of disability categories

Disabilities were considered from subjects' reports. With a 160-page questionnaire, respondents were asked about 1) difficulties in performing activities, 2) restrictions in participations, and 3) environmental factors. Experts (AP, CP, SP) provided the linkage between the WHO-ICF core-set categories for musculoskeletal diseases [13] and the DH survey categories for RMDs (**Table S2**).

### Statistical analysis

The final weighting factors combined design weights and non-response weights. Design weights were the inverse of the sampling fraction, depending on presumed disability severity and geographic area of residence. Probability of non-response was estimated by logistic regression, with age, sex, type of household, marital status, and questions about health and disability as independent variables. Finally, calibration was based on geographic area of residence, age and sex.

For the descriptive analysis, we reported the prevalence of diseases, summarized socio-demographic characteristics and described disabilities by frequencies, means and 95% confidence intervals (95% CIs) estimated using sampling weight.

We assessed the contribution of RMDs at both the individual and societal level. To describe the individual risk of disability with a RMD, multiple regression analysis was used to estimate the strength of association between RMDs and disability categories, controlling for age, sex, number of RMDs and co-morbidities, and educational attainment. Results are expressed as odds ratios (ORs) and 95% CIs.

To assess the contribution of RMDs at the societal level, we used the average attributable fraction (AAF), defined as the expected proportion of disability preventable by the additional elimination of the condition of interest, after adjustment for a random collection of other disorders (here other RMDs and co-morbidities) [17]. The methodology for calculating the AAF was previously described in detail [2]. Briefly, the AAF is considered a relevant statistic for use in co-morbid populations and can be interpreted as follows: an AAF of 20% for OA means that 20% of disability could be avoided by eliminating OA in the population. Because the prevalence of diseases and frequencies of disability vary by age and sex, we computed AAFs by age group (≤20, 20–40, 41–60, 61–80, and >80 years old) and sex.

Statistical analyses involved use of SAS 9.2 (SAS Inst., Cary, NC). Sampling weights were accounted for by specific SAS procedures for handling complex sample designs. AAFs were computed with use of the macro developed by Rückinger et al. [18] with modification to account for sample design [2].

## Results

### Prevalence of RMDs

**Figure 2:**
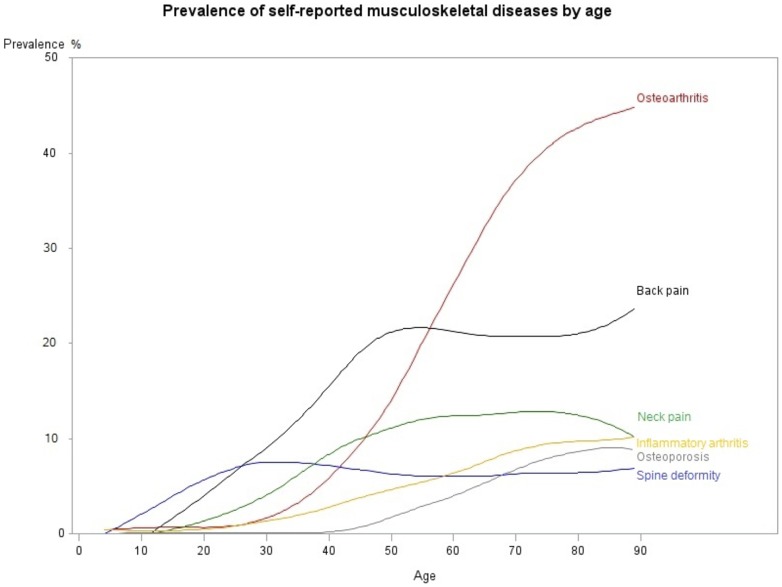
Prevalence of RMDs in France by age.

Overall, 27.7% (95% CI 26.9–28.4%) of the population living in a household reported at least one RMD, corresponding to 17.3 million people. LBP (12.5%, 12.1–13.1%) and OA (12.3%, 11.8–12.7%) were the most prevalent disorders (**Figure 2**). The characteristics of the population reporting RMDs are described in **Table 1**. Type and frequency of RMDs varied by age and sex (**Table S3**). Subjects reporting RMDs were older and had more co-morbidities than those without RMDs (mean age 55.4 years, 54.9–55.9 *vs* 32.8 years, 32.4–33.2), and 59.7% (58.3–61.2%) were women.

**Table 1:** Characteristics of the population reporting rheumatic and musculoskeletal diseases (RMDs).

	Osteoarthritis	Low back pain	Neck pain	Inflammatory arthritis	Spine deformity	Osteoporosis	At least 1 RMD	No RMD
Age (years, mean)	64.2 (63.6–64.8)	53.7 (53.0–54.4)	55.3 (54.4–56.2)	59.8 (58.7–61.0)	45.0 (43.9–46.2)	68.6 (67.6–69.6)	55.4 (54.8–55.9)	32.8 (32.4–33.2)
Sex: women (%)	63.0 (61.1–64.9)	53.5 (51.3–55.7)	68.7 (66.0–71.4)	62.1 (58.4–65.7)	63.3 (59.9–66.8)	91.1 (88.1–94.1)	59.7 (58.3–61.2)	48.5 (47.4–49.6)
Co–morbidity (mean)	2.5 (2.4–2.5)	2.2 (2.2–2.3)	2.7 (2.6–2.8)	2.6 (2.5–2.8)	2.1 (2.0–2.3)	2.8 (2.6–2.9)	2.1 (2.0–2.1)	0.8 (0.8–0.8)
Number of RMDs (mean)	1.9 (1.8–1.9)	1.9 (1.9–2.0)	2.4 (2.3–2.4)	2.2 (2.2–2.3)	2.1 (2.0–2.1)	2.4 (2.3–2.5)	1.5 (1.5–2.1)	–
Educational attainment: no diploma (%)	53.1 (51.2–55.1)	34.8 (32.9–36.8)	37.6 (35.0–40.2)	44.2 (40.7–47.8)	30.0 (27.2–32.9)	51.7 (46.9–56.6)	39.2 (37.8–40.5)	42.8 (41.6–43.7)

Data are % (95% confidence intervals).

### Individual risk of disability with RMDs

Regarding the results of the descriptive analysis (**Table S4**), 21.5% (19.8–23.2%) of subjects reporting OA and 22.5% (19.8–25.3%) reporting inflammatory arthritis had problems walking, compared with 5.3% (4.9–5.7%) of subjects without RMDs. Overall, 16.7% (14.2–19.1%) of subjects reporting inflammatory arthritis had difficulties for shopping (compared with 2.2% (2.1–2.4%) of those without RMD), and 22.3% (19.6–25.1%) for doing housework (compared with 2.6% (2.4–2.8%) of those without RMD). Finally, 49.9% (46.0–53.7%) of subjects reporting osteoporosis had difficulties carrying objects (compared with 15.4% (14.7–16.1%) of those without RMD); 29.8% (26.7–32.9%), needed help from immediate family members (compared with 0.5% (0.4–0.6%) of those without RMD) and 22.7% (19.9–25.4%) from health professionals (compared with 1.8% (1.6–2.0%) of those without RMD). On univariate analysis, subjects with RMDs showed an increased risk of disability in comparison with subjects without (**Table S5**). On multivariate analysis (**Table 2**), inflammatory arthritis was significantly associated with limitation in every activity (from OR 1.4, 1.2–1.8 for walking, to 2.1, 1.5–2.9 for moving around). OA and osteoporosis were associated with difficulties in walking (OR 1.9, 1.7–2.2 and 1.4, 1.1–1.8, respectively) and carrying objects (1.7, 1.5–2.0 and 2.1, 1.6–2.8, respectively). Spine deformity was associated with limitations in changing basic position (1.4, 1.1–1.9) and moving around (1.6, 1.2–2.2). After adjusting for co-morbidities and other RMDs, LBP and neck pain did not increase the risk of limitations in activities, but LBP remained significantly associated with changing jobs because of a health problem (OR 2.2, 1.0–4.5). Regarding environmental factors, inflammatory arthritis and spine deformity were associated with needing more help from immediate family members (1.4, 1.2–1.8 and 1.4, 1.2–1.8, respectively); inflammatory arthritis and osteoporosis were associated with needing more help from health professionals (1.4, 1.1–1.7 and 1.3, 1.1–1.7, respectively).

**Table 2:** Multivariate analysis of the association of disability categories of the WHO-ICF core set for rheumatic and musculoskeletal diseases (RMDs) and RMDs.

Disability category	Osteoarthritis	Low back pain	Neck pain	Inflammatory arthritis	Spine deformity	Osteoporosis	No RMD
Changing basic body position^a^	1.0 (0.8–1.2)	0.7 (0.5–0.9)*	0.6 (0.5–0.8)*	1.7 (1.3–2.4)*	1.4 (1.1–1.9)*	1.0 (0.7–1.3)	1.1 (0.8–1.4)
Lifting and carrying objects^a^	1.7 (1.5–2.0)**	0.6 (0.5–0.7)**	0.5 (0.4–0.6)**	1.7 (1.4–2.1)**	0.7 (0.6–0.8)**	2.1 (1.6–2.8)**	1.0 (0.9–1.2)
Walking^a^	1.9 (1.7–2.2)**	0.6 (0.5–0.7)**	0.5 (0.4–0.6)**	1.4 (1.2–1.8)*	0.7 (0.6–0.9)*	1.4 (1.1–1.8)*	0.9 (0.8–1.0)
Moving around^a^	0.9 (0.7–1.0)	0.6 (0.5–0.8)**	0.6 (0.5–0.8)*	2.1 (1.5–2.9)**	1.6 (1.2–2.2)*	1.2 (0.9–1.5)	1.3 (1.0–1.7)*
Using transportation^a^	1.0 (0.8–1.2)	0.7 (0.6–0.8)*	0.7 (0.5–0.9)*	1.5 (1.2–1.9)*	1.5 (1.2–1.9)*	1.3 (1.0–1.7)*	1.3 (1.0–1.5)*
Driving^a^	0.8 (0.7–1.0)	0.9 (0.7–1.2)	0.8 (0.6–1.1)	1.5 (1.1–2.1)*	1.2 (0.9–1.7)	1.2 (0.9–1.6)	1.3 (1.0–1.6)
Washing oneself^a^	1.0 (0.9–1.2)	0.6 (0.5–0.7)*	0.8 (0.6––1.0)*	1.9 (1.4–2.4)**	1.2 (0.9–1.5)	1.1 (0.9–1.5)	1.2 (1.0–1.5)
Dressing^a^	1.1 (0.9–1.4)	0.7 (0.5–0.8)*	0.7 (0.5–0.9)*	1.7 (1.3–2.3)**	1.2 (0.9–1.6)	1.0 (0.7–1.3)	1.0 (0.8–1.3)
Shopping^a^	0.9 (0.8–1.1)	0.8 (0.7–1.0)*	0.6 (0.5–0.8)**	1.5 (1.2–1.9)*	1.5 (1.2–1.9)*	1.2 (1.0–1.5)	1.0 (0.9–1.2)
Doing housework^a^	1.0 (0.9–1.1)	0.8 (0.7–0.9)*	0.7 (0.6–0.9)*	1.5 (1.2–1.8)*	1.3 (1.1–1.7)*	1.0 (0.8–1.3)	1.0 (0.8–1.2)
Changing job^b^	0.5 (0.2–1.1)	2.2 (1.0–4.5)*	2.8 (0.9–9.2)	0.3 (0.1–1.0)	0.5 (0.1–1.7)	0.5 (0.2–1.4)	0.9 (0.4–2.0)
Community life^b^	1.0 (0.8–1.1)	1.2 (1.0–1.4)*	0.9 (0.7–1.0)	1.1 (0.9–1.4)	0.8 (0.7–1.0)*	1.0 (0.8–1.3)	1.0 (0.9–1.2)
Recreation and leisure^b^	1.1 (0.9–1.4)	0.9 (0.8–1.2)	0.8 (0.6–1.0)*	1.2 (0.9–1.5)	1.1 (0.9–1.4)	0.9 (0.6–1.2)	0.7 (0.6–0.9)*
Help from immediate family^c^	1.0 (0.9–1.1)	0.8 (0.7–0.9)*	0.7 (0.6–0.8)**	1.4 (1.2–1.8)*	1.4 (1.2–1.8)*	1.2 (0.9–1.5)	1.0 (0.9–1.2)
Help from health professionals^c^	1.1 (1.0–1.3)	0.7 (0.6–0.9)*	0.6 (0.5–0.8)**	1.4 (1.1–1.7)*	1.0 (0.8–1.2)	1.3 (1.1–1.7)*	1.1 (0.9–1.3)
Discrimination from the family^c^	0.8 (0.5–1.4)	1.5 (0.9–2.3)	1.0 (0.5–1.7)	0.5 (0.3–1.0)	1.2 (0.7–2.1)	1.1 (0.6–2.2)	1.1 (0.6–1.8)
Discrimination from the society^c^	0.9 (0.7–1.1)	1.1 (0.8–1.4)	0.8 (0.6–1.1)	0.9 (0.7–1.3)	1.4 (1.0–1.9)*	0.7 (0.5–1.0)*	0.8 (0.7–1.1)
Health services delivery^c^	1.0 (0.8–1.2)	1.0 (0.8–1.2)	0.8 (0.7–1.1)	1.1 (0.9–1.5)	1.3 (1.0–1.7)	0.7 (0.5–1.0)*	0.7 (0.6–0.8)*

Data are odds ratios (95% confidence intervals) controlling for age, sex, number of RMDs, number of co-morbidities, and educational attainment. Reference categories are: no osteoarthritis for osteoarthritis, no low back pain for low back pain, no neck pain for neck pain, no inflammatory arthritis for inflammatory arthritis, no spine deformity for spine deformity, no osteoporosis for osteoporosis, and at least 1 RMD for no RMD. ^a^Reference category =  no limitation in activities, ^b^Reference category =  no restriction of participations, ^c^Reference category =  no help, no discrimination, no need of resource.

*p<0.05, **p<0.0001.

### Societal impact of RMDs on disability

**Figure 3:**
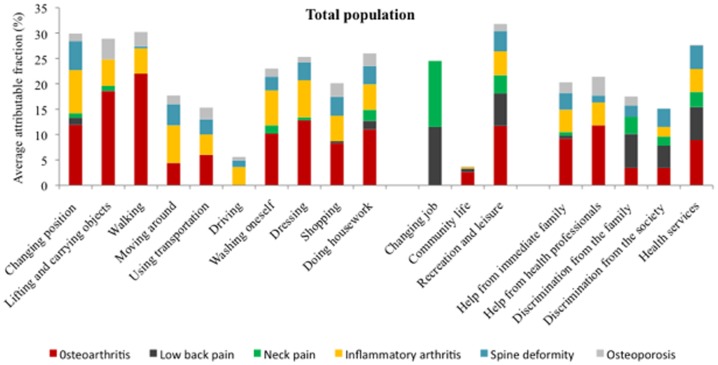
Average attributable fraction (AAF) estimates (%) for disability categories of the core set for RMDs of the WHO-ICF and RMDs from the 2008–2009 Disability-Health Survey in France.

The AAFs for RMDs for disability categories are presented in **Table 3** and **Figure 3**. OA was the main contributor to limitations in activities in the population living in a household, with 22% difficulties in walking, 18.6% difficulties in carrying objects, and 12.8% difficulties in dressing attributable to OA. OA was also a contributor to need for human assistance (9.2% of the need for help from immediate family, 11.8% of the need for help from health professionals, and 8.9% of the need for health service delivery were attributable to OA).

**Table 3:** Average attributable fraction (AAF) estimates (%) for disability categories of the core set for RMDs of the WHO-ICF and RMDs.

	Osteoarthritis	Low back pain	Neck pain	Inflammatory arthritis	Spine deformity	Osteoporosis
Changing basic body position	11.9	1.4	0.9	8.5	5.7	1.5
Lifting and carrying objects	18.6	0.0	1.0	5.2	0.0	4.1
Walking	22.0	0.0	0.0	5.0	0.4	2.8
Moving around	4.4	0.0	0.0	7.4	4.2	1.7
Using transportation	6.0	0.0	0.0	4.0	3.0	2.3
Driving	0.0	0.0	0.0	3.6	1.3	0.7
Washing oneself	10.2	0.0	1.6	6.9	2.7	1.6
Dressing	12.8	0.0	0.6	7.3	3.5	1.1
Shopping	8.3	0.4	0.0	5.0	3.7	2.7
Doing housework	11.0	1.7	2.2	5.0	3.6	2.5
Changing job	0.0	11.5	13.0	0.0	0.0	0.0
Community life	2.6	0.7	0.0	0.3	0.0	0.1
Recreation and leisure	11.7	6.4	3.6	4.7	4.0	1.4
Help from immediate family	9.2	0.6	0.7	4.4	3.3	2.1
Help from health professionals	11.8	0.0	0.0	4.5	1.4	3.7
Discrimination from the family	3.4	6.7	3.4	0.0	2.2	1.8
Discrimination from the society	3.4	4.4	1.8	1.9	3.6	0.0
Health service delivery	8.9	6.5	3.0	4.5	4.6	0.2

Neck pain and LBP were the main contributors to changing jobs because of a health problem (AAF 13% and 11.5%, respectively).

As expected, the impact of RMDs on disabilities was influenced by age categories (**Table S6**). For subjects ≤20 years old, spine deformity was the main contributor to all disability categories (AAF 15.7% for changing basic body position and 13.8% for moving around). For subjects 21–40 years old, inflammatory arthritis was the main contributor (AAF 29.2% for changing basic body position, 23.4% for moving around, 16.5% for dressing, 15% for washing oneself, 21.4% for health service delivery). The impact of LBP on disability categories peaked for subjects 41 to 60 years old (AAF 15.2% for changing job, 10% for changing basic position). The impact of OA on limitations in activities increased by age up to 80 years old (AAF for walking difficulties  = 0% for the age class ≤20 years and 24% for the class 61–80 years).

The overall contribution of RMDs to disability was greater for women than men (for OA: AAF for walking, 24.1% for women *vs* 17.4% for men; for washing oneself, 14.6% for women *vs* 3.8% for men) (**Table S6**). Women were more concerned by OA, inflammatory arthritis (AAF for dressing, 8.8% for women *vs* 5.1% for men) and neck pain (AAF for changing jobs, 26.2% for women *vs* 0.9% for men), whereas the impact of LBP was greater for men (AAF for changing jobs, 10.4% for women *vs* 13.2% for men).

## Discussion

We show for the first time the frequency and impact of RMDs on disability at the individual and societal levels in a developed country, using data representative of the whole non-institutionalized population. Our findings highlight that RMDs are highly prevalent and have a significant impact on limitations in activities and restriction in participations, which suggests the need for more efforts to improve care and research in this field.

One strength of our study is that we assessed disabilities encountered with RMDs from an individual perspective by presenting ORs, which is probably useful for patients and clinicians, as well as a societal perspective by presenting AAFs, which is more useful for a general audience and policymakers. Other strengths of this work are that our results are representative of the population living in a household and are therefore valuable to policymakers. We also considered 18 different disability categories for an overview of disabilities encountered in RMDs from the WHO-ICF perspective [13]. Although the common WHO-ICF core set for RMDs used in this work was not validated for neck pain and spine deformity, the disability categories seem to be relevant, but the results regarding these two disorders may have been underestimated. Finally, co-morbidities are frequent in people reporting chronic diseases [19] and RMDs [20], which highlights the need to consider these in analyses. The use of the AAF provides a validated framework for considering co-morbid situations as compared with other approaches, which may overestimate the potential impact of preventive strategies [2], [17], [18].

LBP and OA were the RMDs most frequently reported. Comparison of disease prevalence with those from previous studies is cautioned because of variations in methodology, cultural context, and definition of diseases [1], [21], [22], but our results agree with results from the 2010 WHO GBD study [1] and are closed to those of a European survey [21]. These frequencies from surveys assessing all chronic conditions are lower than those reported from a Dutch survey assessing specifically RMDs [23]. Although the prevalence of LBP is quite similar in developed and developing countries (particularly in India, China, and Lebanon according to COPCORD surveys results), the prevalence of OA is much more important in developed countries [24].

From an individual perspective, because we used the common WHO-ICF core set for RMDs, our findings allow for a more detailed picture of disabilities encountered with RMDs than do previous studies [4], [25]. Subjects reporting OA were twice more limited in walking than those without OA, but did not report using walking aids more often. This finding should encourage promoting the use of assistive devices such as walking aids and adaptation of the environment for people with OA. Subjects reporting inflammatory arthritis were twice more limited in almost all activities of daily living than those without this disease, but the use of assistive devices was similar in the population with and without inflammatory arthritis. This suggests more emphasis on access to assistive devices and occupational therapy for such people. After we adjusted the analysis for other co-morbidities and RMDs, LBP and neck pain were no longer associated with limitations in activities. One explanation could be that part of people reporting back and neck pain may consider that they have spine OA and therefore reported having OA as a co-morbidity in the survey.

From a societal perspective, our results could help health policymakers develop plans to address and prioritize disabilities in the population living in a household. Regarding the growing prevalence of OA and the walking difficulties associated with this disease, increased emphasis should be placed on accessibility in public places and transportation. Regarding the high contribution of inflammatory arthritis to limitations in activities, efforts are needed to improve and diffuse technical aids by reducing the cost, for example. The impact of LBP on changing jobs and the feeling of being discriminated could be alleviated by population-based information campaigns providing positive messages about back pain, which has been efficacious in improving general beliefs about back pain and influencing medical management in Australia [26]. Spine deformity had the highest impact on disability in subjects ≤20 years old. Therefore, systematic screening for scoliosis and kyphosis by school physicians and general practitioners may need to be promoted. Finally, our results highlighting sex disparities regarding RMDs and associated disabilities, with a greater impact on women than men, suggest that measures to diminish disability may need to differ by sex.

Data of the DH survey are not redundant with those of the recently published 2010 WHO GBD study [1], but probably complementary for two main reasons. First, the WHO GBD study focused on published data, sometimes old, whereas the DH survey was a “snapshot” of the situation in France in 2008. Second, the methods of calculation adopted for the WHO analysis was YLD, which differs from the AAF we used; YLD is the global product of the prevalence of a sequela and its associated disability weight derived from judgments of the general public about health state severity [1], whereas the AAF is based on partitioning disability into a set of risk factors [17] and allow to test different disability categories. Policymakers need a global measurement of disability, such as YLD, to assess general prevalence, but a better understanding of the in-depth impact of diseases may be more relevant to develop a stratified management approach to target health priority. As DH survey is the first survey that provided a detailed description of disabilities at a national level, we cannot compare our results on disability with those of previous European studies.

This study has limitations that are common to this type of survey. The main limitation is that data were self-reported and not physician-confirmed, which is likely to be accurate for disability assessment but may lack accuracy for diagnosis. However, this type of approach is relevant from a public health perspective because many people with chronic illness do not seek a health care provider [22]. A usual confusion between OA and inflammatory arthritis [28] may explain the non-zero impact of OA in subjects ≤20 years old and a potential overestimation of its prevalence. However, the prevalence of physician-diagnosed OA in the United States estimated by the 2003–2005 US National Health Interview Survey was 21.6% [29], which is higher than our prevalence (12.3%). We cannot exclude that part of this difference may be explained by a higher obesity and sedentary rates in the United States than France. Secondly, we could not examine some disabilities, such as pain or fatigue [27], because the survey questionnaire did not measure these symptoms. This situation emphasizes the need to improve and standardize the reporting of data in future disability population-based surveys. We also ignored the frequency of fracture in the population reporting osteoporosis, although this sub-group was probably the only one to experience disability [30]. Another limitation is that information on several RMDs, such as fibromyalgia or shoulder impairments, was missing and might represent a substantial burden [31]. Finally, financial data such as costs induced by RMDs were lacking but would be of interest.

## Conclusions

Even if RMDs are not fatal, they are highly prevalent and disabling, having a significant impact on limitations in activities and restrictions in participations. Our findings may help convince policymakers of the need to focus on RMDs to improve population health. The lack of standardisation in data recording and the absence of detailed national data on disability in other developed and developing countries prevent us from comparing results with other populations, and emphasize the need to support international and national efforts to better address the main challenge of disability associated with RMDs with the increasing aging of the populations.

## Supporting Information

Table S1 **Co-morbidities assessed in the 2008–2009 Disability-Health Survey in France.**
(DOC)

Table S2 **Linkage between the core set of disability categories for rheumatic and musculoskeletal diseases (RMDs) in the World Health Organization's International Classification of Functioning, Disability and Health (WHO-ICF) and questions in the 2008–2009 Disability-Health Survey in France.**
(DOC)

Table S3 **Prevalence of RMDs by sex and age classes from the 2008–2009 Disability-Health Survey in France.**
(DOC)

Table S4 **Frequency of disability categories of the WHO-ICF core set for RMDs from the 2008–2009 Disability-Health Survey in France.**
(DOC)

Table S5 **Unadjusted analysis of the association of disability categories of the WHO-ICF core set for rheumatic and musculoskeletal diseases (RMDs) and RMDs from the 2008–2009 Disability-Health Survey in France.**
(DOC)

Table S6 **Average attributable fraction (AAF) estimates (%) for disability categories of the WHO-ICF core set for RMDs from the 2008–2009 Disability-Health Survey in France by sex and age classes.**
(DOC)
