# Supplementary material for: The Burden of Musculoskeletal Conditions
Source: PLoS One. 2014 Mar 4;9(3):e90633. doi: 10.1371/journal.pone.0090633 (PMC3942474; doi:10.1371/journal.pone.0090633)
Supplement: Table S1 — Co-morbidities assessed in the 2008–2009 Disability-Health Survey in France. (DOC) [file pone.0090633.s001.doc]

Table S1

| **Co-morbidities** | **Included diseases** |
| --- | --- |
| **Cancer** | Cancer (including lymphoid, haematopoietic and related tissue) |
| **Cardiovascular** | Myocardial infarction, angina, stroke, heart failure, lower limb arterial occlusive disease, venous insufficiency and high blood pressure |
| **Dermatological** | Psoriasis, eczema and slough |
| **Digestive** | Ulcer, cirrhosis (and other liver diseases) and food allergies |
| **Endocrine** | Diabetes mellitus, disorders of the thyroid gland and obesity (body mass index ≥ 30 kg/m^2^)^a^ |
| **Neurological** | Headache, epilepsy, dementia, Parkinson’s disease, multiple sclerosis and other unspecified neurological problems |
| **Psychiatric** | Depression, anxiety, autism, schizophrenia, trisomy 21 and other unspecified psychiatric impairments |
| **Respiratory** | Asthma, chronic bronchitis and hay fever |
| **Sensorial** | Eyesight problems ^b^  and hearing loss ^c^ |
| **Sequelae of injury** | Sequelae of injury |
| **Urological** | Urinary incontinence, infection of the urinary tract, lithiasis and prostate adenoma |

This classification followed the 10^th^ International Classification of Diseases.

^a^ Calculated from reported height and weight as weight/height^2^

^b^ Eyesight problems included cataract, strabismus and glaucoma reported in the checklist of the questionnaire and a positive answer to the question: “Do you have any eyesight problems?”

^c^ Hearing loss included a positive answer to the questions: "Are you wearing a hearing aid?” or “Do you have any hearing problems?”
