# Supplementary material for: The Burden of Musculoskeletal Conditions
Source: PLoS One. 2014 Mar 4;9(3):e90633. doi: 10.1371/journal.pone.0090633 (PMC3942474; doi:10.1371/journal.pone.0090633)
Supplement: Table S2 — Linkage between the core set of disability categories for rheumatic and musculoskeletal diseases (RMDs) in the World Health Organization's International Classification of Functioning, Disability and Health (WHO-ICF) and questions in the 2008–2009 Disability-Health Survey in France. (DOC) [file pone.0090633.s002.doc]

**Table S2.**

| **WHO-ICF code** | **WHO-ICF category** | **Disability-Health Survey question** | **Disability category considered for this work** |
| --- | --- | --- | --- |
|  | **Activities and participation** |  |  |
| d410 | Changing basic body position | Can you move from bed and chair? ^1^ | Changing basic body position |
| d415 | Maintaining a body position | *Not available in the survey* | - |
| d430 | Lifting and carrying objects | Can you carry a 5 kg bag during 10 meters without help? ^1^ | Lifting and carrying objects |
| d450 | Walking | Can you walk 500 meters without help? ^1^ | Walking |
| d455 | Moving around | Do you have any difficulty for moving around? ^1^ | Moving around |
| d470 | Using transportation | Do you have any difficulty for using transportation? ^1^ | Using transportation |
| d475 | Driving | Do you drive a car? If not, is it because of a disease or a handicap? ^2^ | Driving |
| d510 | Washing oneself | Do you have any difficulty for washing yourself? ^1^ | Washing oneself |
| d540 | Dressing | Do you have any difficulty for dressing? ^1^ | Dressing |
| d620 | Acquisition of goods and services | Do you have any difficulty for doing shopping? ^1^ | Shopping |
| d640 | Doing housework | Do you have any difficulty for doing housework? ^1^ | Doing housework |
| d770 | Intimate relationships | *Not available in the survey* | - |
| d850 | Remunerative employment | Have you changed job because of a health problem? (if not retired) ^2^ | Changing job |
| d910 | Community life | Do you have any group activity? ^2^ | Community life |
| d920 | Recreation and leisure | Would you like to have more recreation and leisure? If yes, are you restricted because of a disease or a handicap? ^2^ | Recreation and leisure |
|  | **Environmental factors** |  |  |
| e310 | Immediate family | Do you have any help from your immediate family? ^2^ | Help from immediate family |
| e355 | Health professionals | Do you have any help from a professional? ^2^ | Help from health professionals |
| e410 | Individual attitudes of immediate family members | Have you been discriminated by your family? ^2^ | Discrimination from the immediate family |
| e450 | Individual attitudes of health professionals | *Not available in the survey* | *-* |
| e460 | Societal attitudes | Have you been discriminated because of a health problem? ^2^ | Discrimination from the society |
| e580 | Health services, systems and policies | Do you have received any resources from MDPH, COTOREP or CDES? ^2^ | Health service delivery |

Data are % (95% confidence intervals)
