# Supplementary material for: The Burden of Musculoskeletal Conditions
Source: PLoS One. 2014 Mar 4;9(3):e90633. doi: 10.1371/journal.pone.0090633 (PMC3942474; doi:10.1371/journal.pone.0090633)
Supplement: Table S3 — Prevalence of RMDs by sex and age classes from the 2008–2009 Disability-Health Survey in France. (DOC) [file pone.0090633.s003.doc]

Table S3

| Men | ≤20 years | 21-40 years | 41-60 years | 61-80 years | >80 years | Overall men population |
| --- | --- | --- | --- | --- | --- | --- |
| Osteoarthritis | 0.3 (0.1–0.5) | 2.0 (1.3–2.7) | 12.8 (11.5–14.2) | 27.3 (25.0–29.6) | 41.4 (36.2–46.7) | 9.4 (8.8–10.0) |
| Low back pain | 0.7 (0.3–1.1) | 9.4 (7.8–11.0) | 21.3 (19.5–23.2) | 20.0 (17.9–22.2) | 17.0 (13.2–20.8) | 12.1 (11.3–12.8) |
| Neck pain | 0.1 (0.0–0.2) | 2.8 (1.9–3.6) | 7.4 (6.3–8.5) | 8.8 (7.3–10.3) | 9.8 (6.6–12.9) | 4.4 (4.0–4.9) |
| Inflammatory arthritis | 0.3 (0.1–0.4) | 1.3 (0.8–1.8) | 3.8 (3.0–4.5) | 6.8 (5.5–8.2) | 8.2 (5.5–10.9) | 2.7 (2.4–3.1) |
| Spine deformity | 1.4 (0.9–1.9) | 5.8 (4.5–7.1) | 5.5 (4.5–6.5) | 4.4 (3.3–5.4) | 3.7 (2.0–5.4) | 4.2 (3.7–4.7) |
| Osteoporosis | 0.0 (0.0–0.0) | 0.1 (0.0–0.1) | 0.4 (0.2–0.5) | 1.3 (0.6–2.0) | 1.4 (0.2–2.7) | 0.4 (0.2–0.5) |

| Women | ≤20 years | 21-40 years | 41-60 years | 61-80 years | >80 years | Overall women population |
| --- | --- | --- | --- | --- | --- | --- |
| Osteoarthritis | 0.5 (0.1–0.8) | 2.4 (1.7–3.0) | 17.8 (16.3–19.3) | 42.5 (40.1–44.9) | 46.2 (42.6–49.8) | 15.0 (14.3–15.7) |
| Low back pain | 0.9 (0.5–1.4) | 10.0 (8.4–11.5) | 20.1 (18.5–21.8) | 21.7 (19.8–23.6) | 22.4 (19.5–25.3) | 13.0 (12.3–13.8) |
| Neck pain | 0.4 (0.1–0.7) | 6.6 (5.4–7.8) | 14.8 (13.4–16.2) | 15.4 (13.7–17.0) | 13.3 (10.9–15.8) | 9.1 (8.5–9.7) |
| Inflammatory arthritis | 0.2 (0.0–0.3) | 1.5 (1.0–2.0) | 5.8 (5.0–6.7) | 9.8 (8.4–11.1) | 10.2 (8.1.12.2) | 4.2 (3.8–4.5) |
| Spine deformity | 3.4 (2.5–4.3) | 8.5 (7.0–9.9) | 7.4 (6.4–8.4) | 7.8 (6.7–8.9) | 8.6 (6.8–10.5) | 6.8 (6.2–7.4) |
| Osteoporosis | 0.04 (0.0–0.1) | 0.1 (0.0–0.2) | 3.2 (2.5–3.9) | 10.8 (9.4–12.3) | 13.4 (11.1–15.6) | 3.4 (3.1–3.7) |

| Total population | ≤20 years | 21-40 years | 41-60 years | 61-80 years | >80 years | Overall population |
| --- | --- | --- | --- | --- | --- | --- |
| Osteoarthritis | 0.4 (0.2–0.6) | 2.2 (1.7–2.7) | 15.4 (14.4–16.4) | 35.6 (33.9–37.2) | 44.5 (41.6–47.5) | 12.3 (11.8–12.7) |
| Low back pain | 0.8 (0.5–1.1) | 9.7 (8.6–10.8) | 20.7 (19.5–21.9) | 20.9 (19.5–22.4) | 20.5 (18.2–22.8) | 12.5 (12.1–13.1) |
| Neck pain | 0.3 (0.1–0.4) | 4.7 (4.0–5.5) | 11.2 (10.3–12.1) | 12.4 (11.2–13.5) | 12.1 (10.2–14.0) | 6.8 (6.4–7.2) |
| Inflammatory arthritis | 0.2 (0.1–0.3) | 1.4 (1.0–1.8) | 4.8 (4.3–5.4) | 8.4 (7.5–9.4) | 9.5 (7.8–11.1) | 3.5 (3.2–3.7) |
| Spine deformity | 2.4 (1.9–2.9) | 7.2 (6.2–8.1) | 6.4 (5.7–7.2) | 6.2 (5.4–7.0) | 6.9 (5.6–8.3) | 5.5 (5.2–5.9) |
| Osteoporosis | 0.0 (0.0–0.0) | 0.1 (0.0–0.1) | 1.8 (1.5–2.2) | 6.5 (5.6–7.4) | 9.2 (7.7–10.8) | 1.9 (1.7–2.1) |

Data are % (95% confidence intervals)
