# Supplementary material for: The Burden of Musculoskeletal Conditions
Source: PLoS One. 2014 Mar 4;9(3):e90633. doi: 10.1371/journal.pone.0090633 (PMC3942474; doi:10.1371/journal.pone.0090633)
Supplement: Table S4 — Frequency of disability categories of the WHO-ICF core set for RMDs from the 2008–2009 Disability-Health Survey in France. (DOC) [file pone.0090633.s004.doc]

Table S4

| **Disability categories** | **Osteoarthritis** | **Low back pain** | **Neck pain** | **Inflammatory arthritis** | **Spine deformity** | **Osteoporosis** | **No RMD** |
| --- | --- | --- | --- | --- | --- | --- | --- |
| Changing basic body position | 2.8 (2.3–3.3) | 1.6 (1.2–1.9) | 2.3 (1.6–3.0) | 5.4 (4.1–6.8) | 3.0 (2.5–3.6) | 6.3 (5.0–7.5) | 0.6 (0.5–0.6) |
| Lifting and carrying objects | 27.9 (25.9–30.0) | 15.4 (14.0–16.9) | 21.9 (19.6–24.3) | 35.6 (32.2–39.0) | 21.4 (19.2–23.6) | 49.9 (46.0–53.7) | 15.4 (14.7–16.1) |
| Walking | 21.5 (19.8–23.2) | 9.3 (8.3–10.2) | 13.0 (11.2–14.9) | 22.5 (19.8–25.3) | 11.0 (9.8–12.2) | 30.8 (27.6–34.0) | 5.3 (4.9–5.7) |
| Moving around | 4.7 (4.1–5.4) | 1.6 (1.2–1.9) | 2.2 (1.6–2.8) | 7.4 (5.5–9.3) | 3.0 (2.4–3.5) | 8.6 (7.2–10.1) | 0.9 (0.8–1.1) |
| Using transportation | 7.6 (6.6–8.6) | 3.0 (2.5–3.6) | 4.5 (3.5–5.5) | 8.9 (7.1–10.6) | 4.3 (3.6–5.0) | 13.9 (11.9–15.9) | 1.6 (1.4–1.7) |
| Driving | 3.8 (3.1–4.6) | 2.4 (1.8–3.0) | 3.0 (2.2–3.7) | 5.4 (3.7–7.0) | 2.5 (1.9–3.0) | 6.5 (5.0–8.0) | 1.2 (1.1–1.3) |
| Washing oneself | 6.0 (5.3–6.8) | 2.2 (1.7–2.6) | 3.9 (3.1–4.8) | 8.8 (7.0–10.7) | 3.9 (3.3–4.5) | 10.5 (9.0–12.0) | 1.1 (1.0–1.3) |
| Dressing | 5.0 (4.2–5.8) | 2.3 (1.8–2.8) | 3.8 (3.0–4.6) | 8.2 (6.5–10.0) | 4.1 (3.2–4.9) | 8.6 (7.2–10.1) | 1.0 (0.9–1.1) |
| Shopping | 12.3 (11.1–13.5) | 5.7 (5.0–6.5) | 8.3 (7.1–9.6) | 16.7 (14.2–19.1) | 8.3 (7.1–9.5) | 23.9 (21.3–26.6) | 2.2 (2.1–2.4) |
| Doing housework | 18.0 (16.4–19.6) | 8.5 (7.4–9.5) | 13.0 (11.3–14.7) | 22.3 (19.6–25.1) | 12.0 (10.6–13–4) | 32.7 (29.7–35.7) | 2.6 (2.4–2.8) |
| Changing job | 0.4 (0.1–0.8) | 0.8 (0.3–1.3) | 1.7 (0.3–3.0) | 0.2 (0.0–0.4) | 0.4 (0.0–0.9) | 0.4 (0.1–0.7) | 0.6 (0.5–0.8) |
| Community life | 68.7 (66.8–70.6) | 67.1 (65.0–69.2) | 63.9 (61.1–66.7) | 69.4 (66.0–72.7) | 59.8 (56.3–63.2) | 70.8 (66.4–75.3) | 61.6 (60.5–62.6) |
| Recreation and leisure | 7.0 (6.0–8.1) | 6.1 (5.0–7.2) | 8.6 (7.1–10.0) | 11.8 (9.8–13.8) | 9.2 (7.7–10.8) | 13.7 (11.2–16.2) | 1.9 (1.7–2.1) |
| Help from immediate family | 17.0 (15.5–18.6) | 9.0 (7.9–10.0) | 12.7 (11.1–14.3) | 22.3 (10.5–25.0) | 13.1 (11.5–14.6) | 29.8 (26.7–32.9) | 3.7 (3.5–4.0) |
| Help from health professionals | 11.6 (10.4–12.8) | 5.3 (4.4–6.1) | 6.8 (5.4–8.1) | 13.0 (10.9–15.0) | 4.9 (4.1–5.6) | 22.7 (19.9–25.4) | 1.8 (1.6–2.0) |
| Discrimination from the family | 0.6 (0.3–0.9) | 0.7 (0.3–1.1) | 1.0 (0.5–1.6) | 0.7 (0.2–1.2) | 1.2 (0.7–1.6) | 1.4 (0.7–2.1) | 0.5 (0.4–0.6) |
| Discrimination from the society | 3.7 (2.9–4.5) | 5.9 (4.6–7.2) | 6.5 (5.0–8.0) | 7.2 (5.1–9.3) | 10.1 (8.3–12.0) | 5.7 (4.3–7.0) | 4.4 (4.0–4.8) |
| Health service delivery | 4.6 (3.9–5.3) | 6.2 (5.2–7.1) | 8.0 (6.6–9.3) | 10.4 (8.5–12.2) | 9.5 (8.2–10.9) | 9.0 (7.3–10.6) | 2.3 (2.1–2.5) |

Data are % (95% confidence intervals)
