# Supplementary material for: The Burden of Musculoskeletal Conditions
Source: PLoS One. 2014 Mar 4;9(3):e90633. doi: 10.1371/journal.pone.0090633 (PMC3942474; doi:10.1371/journal.pone.0090633)
Supplement: Table S5 — Unadjusted analysis of the association of disability categories of the WHO-ICF core set for rheumatic and musculoskeletal diseases (RMDs) and RMDs from the 2008–2009 Disability-Health Survey in France. (DOC) [file pone.0090633.s005.doc]

Table S5.

| **Disability categories** | **Osteoarthritis** | **Low back pain** | **Neck pain** | **Inflammatory arthritis** | **Spine deformity** | **Osteoporosis** | **No RMD** |
| --- | --- | --- | --- | --- | --- | --- | --- |
| Changing basic body position^a^ | 6.2 (5.2–7.3)** | 3.1 (2.6–3.7)** | 3.6 (2.9–4.4)** | 6.9 (5.5–8.7)** | 3.5 (2.8–4.3)** | 5.9 (4.6–7.5)** | 0.2 (0.2–0.2)** |
| Lifting and carrying objects^a^ | 3.0 (2.8–3.3)** | 1.5 (1.4–1.6)** | 1.9 (1.7–2.2)** | 3.3 (2.8–3.8)** | 1.5 (1.3–1.7)** | 4.7 (3.9–5.8)** | 0.6 (0.5–0.6)** |
| Walking^a^ | 5.5 (5.0–6.1)** | 2.1 (1.9–2.3)** | 2.3 (2.1–2.7)** | 4.2 (3.6–4.9)** | 1.8 (1.5–2.0)** | 5.3 (4.4–6.4)** | 0.3 (0.3–0.3)** |
| Moving around^a^ | 5.0 (4.3–5.8)** | 2.0 (1.7–2.3)** | 2.4 (1.9–2.9)** | 5.8 (4.6–7.3)** | 2.4 (2.0–3.0)** | 5.9 (4.7–7.4)** | 0.2 (0.2–0.3)** |
| Using transportation^a^ | 5.1 (4.5–5.8)** | 2.0 (1.7–2.3)** | 2.4 (2.0–2.9)** | 4.4 (3.6–5.3)** | 2.1 (1.8–2.6)** | 6.2 (5.0–7.6)** | 0.3 (0.2–0.3)** |
| Driving^a^ | 3.4 (2.9–4.0)** | 2.0 (1.7–2.4)** | 2.1 (1.7–2.6)** | 3.7 (2.8–4.8)** | 1.7 (1.3–2.1)** | 4.0 (3.0–5.2)** | 0.3 (0.3–0.4)** |
| Washing oneself^a^ | 5.9 (5.1–6.7)** | 2.3 (2.0–2.6)** | 3.2 (2.7–3.8)** | 6.0 (4.9–7.2)** | 2.4 (2.0–2.8)** | 5.7 (4.7–7.0)** | 0.2 (0.2–0.3)** |
| Dressing^a^ | 5.9 (5.1–6.8)** | 2.7 (2.3–3.1)** | 3.2 (2.7–3.8)** | 6.0 (4.9–7.4)** | 2.7 (2.2–3.3)** | 5.1 (4.1–6.3)** | 0.2 (0.2–0.2)** |
| Shopping^a^ | 5.9 (5.4–6.6)** | 2.7 (2.4–3.0)** | 3.0 (2.6–3.4)** | 5.3 (4.5–6.3)** | 2.6 (2.2–3.1)** | 7.3 (6.1–8.8)** | 0.2 (0.2–0.2)** |
| Doing housework^a^ | 7.4 (6.7–8.2)** | 3.2 (2.9–3.6)** | 4.0 (3.5–4.5)** | 6.1 (5.3–7.1)** | 2.9 (2.5–3.3)** | 8.3 (6.9–10.0)** | 0.2 (0.1–0.2)** |
| Changing job ^b^ | 0.7 (0.4–1.3) | 1.5 (0.9–2.7) | 1.9 (0.9–4.2) | 0.4 (0.1–1.2) | 0.6 (0.2–1.9) | 0.6 (0.2–1.4) | 0.9 (0.6–1.5) |
| Community life ^b^ | 1.3 (1.2–1.5)* | 1.2 (1.1–1.4)* | 1.1 (0.9–1.2) | 1.4 (1.2–1.6)* | 0.9 (0.8–1.0) | 1.4 (1.2–1.8)* | 0.8 (0.8–0.9)** |
| Recreation and leisure ^b^ | 4.5 (3.9–5.1) | 3.6 (3.1–4.1) | 4.2 (3.6–4.9) | 4.7 (4.0–5.7) | 3.4 (2.8–4.1) | 4.3 (3.4–5.5) | 0.2 (0.2–0.2)** |
| Help from immediate family^c^ | 5.4(4.9–6.0)** | 2.7 (2.4–3.0)** | 3.1 (2.8–3.6)** | 5.0 (4.3–5.8)** | 2.6 (2.3–3.1)** | 6.2 (5.2–7.5)** | 0.2 (0.2–0.2)** |
| Help from health professionals^c^ | 7.0 (6.2–7.9)** | 2.5 (2.2–2.8)** | 2.8 (2.4–3.2)** | 5.1 (4.3–6.0)** | 2.0 (1.7–2.3)** | 8.4 (6.9–10.2)** | 0.2 (0.2–0.2)** |
| Discrimination from the family ^c^ | 1.8 (1.2–2.7)* | 2.4 (1.6–3.4)** | 2.6 (1.8–3.9)** | 1.7 (1.0–2.7) | 2.5 (1.6–3.8)** | 2.5 (1.4–4.3)* | 0.6 (0.4–0.8)* |
| Discrimination from the society ^c^ | 1.3 (1.1–1.5)* | 1.7 (1.5–2.0)** | 1.9 (1.6–2.3)** | 1.8 (1.4–2.3)** | 2.4 (1.9–2.9)** | 1.2 (0.9–1.5) | 0.7 (0.6–0.8)** |
| Health services delivery ^c^ | 3.2 (2.8–3.6)** | 3.1 (2.7–3.5)** | 3.5 (3.0–4.0)** | 3.9 (3.3–4.7)** | 3.3 (2.8–3.9)** | 2.7 (2.1–3.3)** | 0.3 (0.3–0.3)** |

Data are odds ratios (95% confidence intervals).

Reference categories are: no osteoarthritis for osteoarthritis, no low back pain for low back pain, no neck pain for neck pain, no inflammatory arthritis for inflammatory arthritis, no spine deformity for spine deformity, no osteoporosis for osteoporosis, and at least 1 RMD for no RMD.

^a^Reference category= no limitation in activities, ^b^Reference category= no restriction of participations, ^c^ Reference category= no help, no discrimination, no need of resource

* p<0.05, ** p<0.0001
