# Supplementary material for: The Burden of Musculoskeletal Conditions
Source: PLoS One. 2014 Mar 4;9(3):e90633. doi: 10.1371/journal.pone.0090633 (PMC3942474; doi:10.1371/journal.pone.0090633)
Supplement: Table S6 — Average attributable fraction (AAF) estimates (%) for disability categories of the WHO-ICF core set for RMDs from the 2008–2009 Disability-Health Survey in France by sex and age classes. (DOC) [file pone.0090633.s006.doc]

Table S6

Men

|  | **Osteoarthritis** | **Low back pain** | **Neck pain** | **Inflammatory arthritis** | **Spine deformity** | **Osteoporosis** |
| --- | --- | --- | --- | --- | --- | --- |
| Changing basic body position | 6.6 | 0.0 | 0.5 | 7.3 | 4.6 | 1.3 |
| Lifting and carrying objects | 12.5 | 0.0 | 0.9 | 2.4 | 0.0 | 1.2 |
| Walking | 17.6 | 0.0 | 0.2 | 2.8 | 0.3 | 0.7 |
| Moving around | 0.0 | 0.0 | 0.0 | 4.6 | 4.0 | 1.1 |
| Using transportation | 2.5 | 0.0 | 0.0 | 0.0 | 2.4 | 0.7 |
| Driving | 1.0 | 0.0 | 0.0 | 0.0 | 0.6 | 0.0 |
| Washing oneself | 3.8 | 0.0 | 1.7 | 5.4 | 3.3 | 0.4 |
| Dressing | 6.7 | 0.0 | 3.1 | 5.1 | 2.5 | 0.6 |
| Shopping | 3.2 | 0.0 | 0.0 | 3.3 | 2.7 | 1.3 |
| Doing housework | 4.7 | 0.0 | 1.6 | 1.8 | 2.4 | 0.9 |
| Changing job | 0.0 | 13.2 | 0.9 | 0.0 | 0.0 | 0.0 |
| Community life | 1.1 | 1.8 | 0.0 | 0.5 | 0.0 | 0.2 |
| Recreation and leisure | 8.5 | 5.0 | 2.6 | 4.1 | 0.8 | 1.1 |
| Help from immediate family | 4.7 | 0.0 | 0.0 | 4.5 | 2.0 | 0.8 |
| Help from health professionals | 5.0 | 0.0 | 0.0 | 1.1 | 0.8 | 0.5 |
| Discrimination from the family | 4.8 | 7.4 | 0.0 | 0.0 | 1.0 | 0.0 |
| Discrimination from the society | 3.0 | 2.8 | 0.8 | 0.6 | 2.0 | 0.6 |
| Health service delivery | 8.0 | 7.7 | 1.4 | 3.5 | 4.3 | 0.2 |

Women

|  | **Osteoarthritis** | **Low back pain** | **Neck pain** | **Inflammatory arthritis** | **Spine deformity** | **Osteoporosis** |
| --- | --- | --- | --- | --- | --- | --- |
| Changing basic body position | 15.6 | 2.8 | 1.5 | 9.1 | 6.3 | 1.2 |
| Lifting and carrying objects | 20.5 | 2.4 | 0.0 | 6.8 | 0.2 | 3.9 |
| Walking | 24.1 | 1.0 | 0.0 | 6.4 | 0.3 | 3.4 |
| Moving around | 6.9 | 0.0 | 0.0 | 8.8 | 3.9 | 0.3 |
| Using transportation | 6.8 | 0.0 | 0.0 | 6.6 | 2.9 | 1.8 |
| Driving | 0.0 | 2.6 | 0.0 | 7.6 | 1.7 | 1.4 |
| Washing oneself | 14.6 | 0.0 | 1.7 | 7.8 | 2.2 | 1.7 |
| Dressing | 18 | 1.0 | 0.0 | 8.8 | 4.4 | 1.6 |
| Shopping | 9.1 | 3.1 | 0.0 | 5.8 | 3.9 | 1.5 |
| Doing housework | 12.2 | 3.6 | 1.5 | 6.5 | 3.8 | 1.4 |
| Changing job | 0.0 | 10.4 | 26.2 | 0.0 | 0.0 | 0.0 |
| Community life | 2.9 | 0.8 | 0.0 | 0.6 | 0.0 | 0.3 |
| Recreation and leisure | 13.6 | 7.8 | 4.0 | 4.9 | 6.2 | 1.2 |
| Help from immediate family | 10.9 | 3.4 | 0.3 | 4.2 | 3.9 | 1.7 |
| Help from health professionals | 13.5 | 2.2 | 0.0 | 6.0 | 1.3 | 3.2 |
| Discrimination from the family | 3.7 | 6.1 | 7.1 | 0.7 | 3.0 | 2.7 |
| Discrimination from the society | 4.0 | 5.8 | 2.8 | 3.1 | 5.0 | 0.0 |
| Health service delivery | 10.4 | 3.8 | 6.4 | 5.3 | 5.0 | 1.3 |

Age class ≤20 years

|  | **Osteoarthritis** | **Low back pain** | **Neck pain** | **Inflammatory arthritis** | **Spine deformity** | **Osteoporosis** |
| --- | --- | --- | --- | --- | --- | --- |
| Changing basic body position | 5.0 | 0.0 | 0.0 | 9.8 | 15.7 | 0.0 |
| Lifting and carrying objects | 0.0 | 0.0 | 0.0 | 0.3 | 0.0 | 0.0 |
| Walking | 0.0 | 0.0 | 0.0 | 1.0 | 0.0 | 0.0 |
| Moving around | 8.5 | 0.0 | 0.0 | 11.1 | 13.8 | 0.0 |
| Using transportation | 4.3 | 0.0 | 0.0 | 6.6 | 9.4 | 0.0 |
| Driving | 6.0 | 4.4 | 0.0 | 11 | 7.1 | 0.0 |
| Washing oneself | 3.4 | 0.0 | 0.0 | 5.7 | 6.9 | 0.0 |
| Dressing | 3.3 | 0.0 | 0.0 | 6.7 | 15.0 | 0.0 |
| Shopping | 5.1 | 0.4 | 0.0 | 7.2 | 9.3 | 0.0 |
| Doing housework | 6.8 | 2.9 | 0.0 | 6.2 | 12.0 | 0.0 |
| Changing job | 0.0 | 0.0 | 0.0 | 0.0 | 0.0 | 0.0 |
| Community life | 0.2 | 0.1 | 0.0 | 0.0 | 0.0 | 0.0 |
| Recreation and leisure | 8.4 | 2.3 | 0.3 | 2.4 | 8.0 | 0.0 |
| Help from immediate family | 1.0 | 0.2 | 0.0 | 2.7 | 4.6 | 0.0 |
| Help from health professionals | 3.3 | 0.0 | 0.0 | 3.5 | 1.5 | 0.0 |
| Discrimination from the family | 0.0 | 0.0 | 0.0 | 0.0 | 0.0 | 0.0 |
| Discrimination from the society | 0.7 | 1.1 | 0.0 | 2.2 | 4.0 | 0.0 |
| Health service delivery | 3.4 | 0.0 | 0.0 | 5.3 | 7.7 | 0.0 |

Age class 21-40 years

|  | **Osteoarthritis** | **Low back pain** | **Neck pain** | **Inflammatory arthritis** | **Spine deformity** | **Osteoporosis** |
| --- | --- | --- | --- | --- | --- | --- |
| Changing basic body position | 3.5 | 1.0 | 0.0 | 29.2 | 9.3 | 6.8 |
| Lifting and carrying objects | 9.2 | 9.0 | 4.3 | 7.5 | 7.9 | 5.8 |
| Walking | 7.5 | 0.0 | 7.9 | 11.2 | 3.6 | 4.2 |
| Moving around | 0.4 | 0.0 | 0.0 | 23.4 | 3.1 | 6.7 |
| Using transportation | 6.9 | 0.0 | 0.5 | 0.0 | 6.6 | 2.6 |
| Driving | 3.4 | 0.0 | 2.6 | 0.0 | 4.5 | 2.0 |
| Washing oneself | 5.4 | 0.0 | 0.0 | 15.0 | 6.1 | 5.9 |
| Dressing | 16.2 | 3.6 | 0.0 | 16.5 | 15.1 | 2.9 |
| Shopping | 8.5 | 1.9 | 1.0 | 8.5 | 10.1 | 5.8 |
| Doing housework | 4.4 | 4.9 | 6.3 | 4.8 | 6.0 | 4.5 |
| Changing job | 0.0 | 0.0 | 39.2 | 0.0 | 7.2 | 0.0 |
| Community life | 0.4 | 1.6 | 0.0 | 0.0 | 0.0 | 0.2 |
| Recreation and leisure | 12.6 | 7.9 | 3.0 | 3.1 | 5.4 | 1.7 |
| Help from immediate family | 15.4 | 2.8 | 3.5 | 15.6 | 7.0 | 5.8 |
| Help from health professionals | 11.5 | 3.1 | 0.0 | 0.0 | 0.9 | 2.7 |
| Discrimination from the family | 2.6 | 2.6 | 2.4 | 2.5 | 3.5 | 1.4 |
| Discrimination from the society | 5.0 | 2.8 | 0.0 | 1.3 | 3.9 | 0.7 |
| Health service delivery | 12.2 | 1.6 | 3.9 | 21.4 | 0.7 | 5.5 |

Age class 41-60 years

|  | **Osteoarthritis** | **Low back pain** | **Neck pain** | **Inflammatory arthritis** | **Spine deformity** | **Osteoporosis** |
| --- | --- | --- | --- | --- | --- | --- |
| Changing basic body position | 16.5 | 10.0 | 1.0 | 12.0 | 3.9 | 2.7 |
| Lifting and carrying objects | 15.6 | 5.5 | 4.8 | 9.3 | 2.8 | 3.1 |
| Walking | 21.5 | 4.8 | 0.0 | 8.1 | 0.8 | 1.9 |
| Moving around | 9.0 | 0.0 | 0.0 | 7.3 | 3.4 | 2.6 |
| Using transportation | 7.6 | 0.0 | 0.0 | 5.2 | 0.8 | 4.0 |
| Driving | 0.0 | 0.0 | 0.0 | 5.3 | 2.1 | 1.7 |
| Washing oneself | 11.0 | 0.0 | 5.9 | 11.2 | 1.1 | 3.6 |
| Dressing | 12.8 | 2.3 | 5.2 | 10.7 | 0.8 | 2.3 |
| Shopping | 9.9 | 1.1 | 1.5 | 6.7 | 0.9 | 3.3 |
| Doing housework | 10.2 | 2.7 | 6.0 | 8.2 | 2.7 | 3.2 |
| Changing job | 0.0 | 15.2 | 5.0 | 0.0 | 0.0 | 0.0 |
| Community life | 0.0 | 0.6 | 0.0 | 0.0 | 0.0 | 0.4 |
| Recreation and leisure | 13.0 | 7.8 | 6.7 | 5.7 | 3.3 | 2.6 |
| Help from immediate family | 9.3 | 3.5 | 2.4 | 7.5 | 2.4 | 2.6 |
| Help from health professionals | 1.3 | 0.0 | 1.5 | 6.0 | 0.8 | 6.3 |
| Discrimination from the family | 6.0 | 8.8 | 0.3 | 0.0 | 0.3 | 2.2 |
| Discrimination from the society | 6.5 | 4.4 | 3.9 | 3.1 | 0.5 | 0.2 |
| Health service delivery | 12.7 | 7.2 | 0.8 | 4.6 | 4.7 | 1.0 |

Age class 61-80 years

|  | **Osteoarthritis** | **Low back pain** | **Neck pain** | **Inflammatory arthritis** | **Spine deformity** | **Osteoporosis** |
| --- | --- | --- | --- | --- | --- | --- |
| Changing basic body position | 11.0 | 0.3 | 1.0 | 7.2 | 4.6 | 2.7 |
| Lifting and carrying objects | 20.9 | 3.2 | 1.0 | 5.0 | 2.2 | 3.6 |
| Walking | 24.0 | 2.1 | 0.0 | 4.6 | 1.7 | 1.6 |
| Moving around | 4.2 | 0.0 | 0.0 | 9.1 | 3.4 | 1.5 |
| Using transportation | 2.5 | 0.0 | 0.0 | 9.2 | 2.4 | 2.1 |
| Driving | 0.0 | 3.8 | 0.0 | 5.7 | 0.0 | 1.5 |
| Washing oneself | 11.0 | 0.0 | 0.0 | 8.2 | 3.8 | 1.4 |
| Dressing | 15.0 | 1.9 | 0.2 | 5.6 | 1.0 | 0.9 |
| Shopping | 11.9 | 0.7 | 0.0 | 6.6 | 2.6 | 3.5 |
| Doing housework | 15.4 | 0.8 | 0.2 | 5.2 | 3.5 | 2.9 |
| Changing job | 0.0 | 49.5 | 6.2 | 0.0 | 0.0 | 0.0 |
| Community life | 0.0 | 0.0 | 0.0 | 0.9 | 0.4 | 0.0 |
| Recreation and leisure | 8.0 | 3.9 | 1.8 | 6.6 | 3.4 | 1.9 |
| Help from immediate family | 11.8 | 0.0 | 0.2 | 4.6 | 1.7 | 2.9 |
| Help from health professionals | 19.3 | 1.1 | 0.0 | 5.9 | 2.4 | 3.7 |
| Discrimination from the family | 16.7 | 4.8 | 13.5 | 0.0 | 8.0 | 7.4 |
| Discrimination from the society | 0.7 | 5.1 | 2.9 | 0.0 | 7.5 | 3.2 |
| Health service delivery | 8.7 | 3.9 | 5.2 | 2.7 | 5.3 | 0.0 |

Age class >80 years

|  | **Osteoarthritis** | **Low back pain** | **Neck pain** | **Inflammatory arthritis** | **Spine deformity** | **Osteoporosis** |
| --- | --- | --- | --- | --- | --- | --- |
| Changing basic body position | 10.5 | 0.0 | 1.1 | 3.3 | 5.8 | 0.0 |
| Lifting and carrying objects | 8.5 | 1.6 | 0.3 | 2.1 | 2.2 | 3.5 |
| Walking | 14.3 | 0.0 | 1.9 | 1.9 | 2.7 | 3.0 |
| Moving around | 4.8 | 0.0 | 0.8 | 4.4 | 3.9 | 1.8 |
| Using transportation | 9.3 | 0.0 | 1.3 | 0.0 | 2.4 | 2.3 |
| Driving | 0.0 | 0.5 | 1.5 | 1.2 | 0.2 | 0.0 |
| Washing oneself | 10.1 | 0.0 | 2.5 | 2.9 | 7.6 | 7.9 |
| Dressing | 8.2 | 0.0 | 0.0 | 5.1 | 2.8 | 0.7 |
| Shopping | 4.0 | 0.9 | 0.9 | 1.6 | 3.0 | 1.8 |
| Doing housework | 10.4 | 3.1 | 1.7 | 3.3 | 2.1 | 2.3 |
| Changing job | 0.0 | 0.0 | 0.0 | 0.0 | 0.0 | 0.0 |
| Community life | 3.1 | 0.0 | 0.0 | 0.6 | 0.0 | 0.4 |
| Recreation and leisure | 15.2 | 7.2 | 1.2 | 2.5 | 0.0 | 2.4 |
| Help from immediate family | 5.7 | 1.2 | 0.3 | 0.9 | 3.7 | 0.3 |
| Help from health professionals | 9.3 | 1.7 | 1.0 | 4.1 | 0.0 | 3.8 |
| Discrimination from the family | 2.6 | 0.0 | 0.0 | 0.0 | 0.0 | 2.6 |
| Discrimination from the society | 27.2 | 7.3 | 0.0 | 20.0 | 14.1 | 0.0 |
| Health service delivery | 0.9 | 5.5 | 3.6 | 3.5 | 10.5 | 0.9 |
